# Large variations in hospital pricing for standard procedures revealed

**DOI:** 10.1186/s13104-022-06014-2

**Published:** 2022-04-05

**Authors:** Chan Shen, Jennifer L. Moss

**Affiliations:** 1 Department of Surgery, College of Medicine, The Pennsylvania State University, Hershey, PA USA; 2 Department of Public Health Sciences, College of Medicine, The Pennsylvania State University, Hershey, PA USA; 3 Department of Family and Community Medicine, College of Medicine, The Pennsylvania State University, Hershey, PA USA

**Keywords:** Hospital price transparency, Pricing variations, Insurance plan

## Abstract

**Objective:**

The CMS mandated hospital price transparency reporting on January 1, 2021 aiming to empower patients, enhance market competition, and curtail healthcare costs in the US. We aimed to characterize variability in hospital pricing reported by 1982 hospitals on six standard procedures (including abdominal ultrasound, diagnostic colonoscopy, kidney function blood test panel, knee arthroscopic cartilage removal, magnetic resonance imaging scan of brain, and pelvis computed tomography scan with contrast), with a particular focus on variations in pricing by insurance plan type.

**Results:**

We found substantial heterogeneity across insurance plan types. The minimum number of prices reported was 18,679 for knee arthroscopic cartilage removal (reported by 908 hospitals, average = 21 prices/hospital), while the maximum number of prices reported was 44,921 for abdominal ultrasound (reported by 1861 hospitals, average = 24 prices/hospital). In general, reported hospital pricing was highest for the list price, followed by cash price and prices negotiated with commercial insurance plans. Government insurance, including Medicare, Medicaid and Veterans/Tricare plans, had much lower prices. However, prices were very heterogeneous with substantial overlaps between pricing for all plan types. The coefficients of variation for all procedures exceeded 100%, ranging from 106% for knee arthroscopic cartilage removal to 397% for kidney function blood test panel.

**Supplementary Information:**

The online version contains supplementary material available at 10.1186/s13104-022-06014-2.

## Introduction

Healthcare costs are substantially higher in the US compared to other developed countries [1]. These differences could be due to a lack of transparency coupled with large variations in hospital pricing for standard procedures, which hinder market competition [2, 3]. Costs are a substantial barrier for patients to make informed decisions on healthcare. The Centers for Medicare and Medicaid Services (CMS) implemented a new rule on January 1, 2021, which mandates that hospitals disclose their pricing in an attempt to empower patients, enhance market competition, and curtail healthcare costs in the US [4, 5].

We aimed to characterize variability in hospital pricing reported by 1982 hospitals on six standard procedures, with a particular focus on variations in pricing by insurance plan type. This preliminary analysis can generate hypotheses for future research studies designed to predict hospital pricing, with long-term implications for interventions and policies to improve transparency, reduce variability, and improve health and financial outcomes for US patients.

## Main text

We examined hospital pricing data for 1982 hospitals on six standard procedures based on the Current Procedural Terminology (CPT) codes including: abdominal ultrasound (CPT code: 76700), diagnostic colonoscopy (CPT code: 45378), kidney function blood test panel (CPT code: 80069), knee arthroscopic cartilage removal (CPT code: 29881), magnetic resonance imaging (MRI) scan of brain (CPT code: 70553), and pelvis computed tomography (CT) scan with contrast (CPT code: 72193), based on data provided by Turquoise Health (https://turquoise.health/researchers).

Each hospital may report several distinct prices (average 23 prices per procedure) based on types of insurance plans, which we group into six categories as described below. If the plan name started with “cash” or “list,” it was considered cash price or list price respectively; if the plan name included “Medicare” or “Medicaid,” it was considered Medicare or Medicaid plans respectively; Veterans/Tricare plan was identified based on the keywords: “Veterans,” “Tricare,” or “VA”; and all others were considered commercial/other. To demonstrate how the prices correspond to medical bills patients receive, we provided an example bill in Additional file 1: Table S1. On this example bill for an ultrasound, the “Charges” column shows the list price ($1036); subtracting “Insurance Adjustment” from the “Charges” yields the price negotiated with insurance ($1036–$923 = $113) for Medicaid; “Insurance Payment” is the amount paid by insurance ($110); and patient’s out-of-pocket cost ($3) is in the “Patient Balance” column.

We generated box plots by procedure and plan type, and provided the mean, standard deviation (SD), median, coefficient of variation (CV, defined as SD/mean), interquartile range (IQR) and range. Because pricing data were not normally distributed, we used Kruskal–Wallis test to examine the subgroup differences in hospital pricing by insurance plan type. The p-values are presented in Table 1. To make the boxplots more readable and visually informative, we cut off extreme values above 10 times the average IQR away from the average 1st quartile across groups from the plots (i.e. clip factor of 10 was applied). All statistical analyses were conducted in SAS 9.4 (SAS Institute, Cary NC).

**Table 1:** Prices by type of insurance plan

		Plan type							
		List price	Cash price	Medicare	Medicaid	Veterans/Tricare	Commercial/Other	Total	P-value
Abdominal Ultrasound (reported by 1861 hospitals)		(N = 1447)	(N = 2003)	(N = 5083)	(N = 2137)	(N = 819)	(N = 33,432)	(N = 44,921)	
									< .0001
	N	1447	2003	5083	2137	819	33,432	44,921	
	Mean	1257.2	866.4	268.5	198.7	269.4	671.3	623.5	
	SD	1265.96	1413.73	413.85	590.68	349.79	889.45	900.02	
	Median	1036	617.6	118.4	113	112.8	532.5	444	
	CV	101%	163%	154%	297%	130%	132%	144%	
	IQR	738, 1557	321, 978	107, 199	70, 193	103, 302	232, 862	160, 833	
	Range	23, 39,796	0, 42,640	2, 2589	0, 24,141	44, 2845	0, 37,806	0, 42,640	
Diagnostic Colonoscopy (reported by 1279 hospitals)		(N = 877)	(N = 1181)	(N = 3771)	(N = 1350)	(N = 572)	(N = 21,532)	(N = 29,283)	
									< .0001
	N	877	1181	3771	1350	572	21,532	29,283	
	Mean	4822.6	2940.2	907.4	674.3	959.8	2510.5	2275.7	
	SD	8872.55	5421.44	611.37	633.9	1012.69	3101.48	3368.21	
	Median	2706	1734.9	764	480.8	719.1	1913	1541.9	
	CV	184%	184%	67%	94%	106%	124%	148%	
	IQR	1585, 4249	1009, 2958	693, 895	375, 762	684, 794	1033, 3284	769, 2864	
	Range	1, 93,050	0, 69,788	100, 9014	0, 7528	102, 13,212	0, 330,372	0, 330,372	
Kidney Function Blood Test Panel (reported by 1815 hospitals)		(N = 1398)	(N = 1911)	(N = 4118)	(N = 1685)	(N = 631)	(N = 30,685)	(N = 40,428)	
									< .0001
	N	1398	1911	4118	1685	631	30,685	40,428	
	Mean	253.8	191.5	43.7	35.8	52.6	143	133.1	
	SD	733.74	569.6	315.64	234.88	164.1	550.96	528.43	
	Median	161	85.2	8.7	9.4	8.7	73	57.9	
	CV	289%	297%	722%	656%	312%	385%	397%	
	IQR	89, 280	42, 157	9, 10	8, 17	9, 47	21, 153	12, 139	
	Range	3, 18,435	0, 12,904	2, 6490	1, 6012	3, 2276	0, 17,513	0, 18,435	
Knee arthroscopic cartilage removal (reported by 908 hospitals)		(N = 519)	(N = 772)	(N = 2261)	(N = 730)	(N = 455)	(N = 13,942)	(N = 18,679)	
									< .0001
	N	519	772	2261	730	455	13,942	18,679	
	Mean	12,104	7853.5	2769.8	2252.1	3343.3	7872.6	7041.7	
	SD	11,226.32	6895.23	1499.78	12,082.02	3711.02	7264.83	7473.17	
	Median	9156.1	5541.4	2615.9	1486	2527.9	5738.4	4437	
	CV	93%	88%	54%	536%	111%	92%	106%	
	IQR	4499, 15,796	3009, 10,575	2377, 3029	823, 2148	2414, 2816	2707, 11,119	2482, 9302	
	Range	210, 101,792	0, 40,717	100, 31,294	0, 324,864	215, 58,390	0, 226,444	0, 324,864	
MRI scan of brain (reported by 1846 hospitals)		(N = 1398)	(N = 1975)	(N = 4971)	(N = 2047)	(N = 783)	(N = 31,902)	(N = 43,076)	
									< .0001
	N	1398	1975	4971	2047	783	31,902	43,076	
	Mean	4979.9	3294.3	750.8	698.2	1011.3	2518.5	2316	
	SD	2909.51	5889.33	752.65	849.78	1380.84	2334.74	2603.44	
	Median	4370.1	2482.2	417.8	445.1	384.2	1943.1	1620	
	CV	58%	179%	100%	122%	137%	93%	112%	
	IQR	3114, 6112	1388, 3886	366, 823	293, 732	347, 1148	853, 3551	576, 3333	
	Range	18, 26,472	0, 231,400	14, 8879	0, 8982	90, 11,255	0, 57,090	0, 231,400	
Pelvis CT Scan with Contrast (reported by 1776 hospitals)		(N = 1366)	(N = 1900)	(N = 4383)	(N = 1748)	(N = 738)	(N = 29,987)	(N = 40,122)	
									< .0001
	N	1366	1900	4383	1748	738	29,987	40,122	
	Mean	2875.7	1973.3	401.6	382.7	558.1	1459.8	1353.2	
	SD	1783.58	3003.03	650.86	516.16	847.98	1909.08	1894.02	
	Median	2478.5	1381.6	199.4	236.3	186.5	1192.9	1027.8	
	CV	62%	152%	162%	135%	152%	131%	140%	
	IQR	1754, 3366	822, 2223	177, 446	146, 400	168, 641	500, 1969	350, 1871	
	Range	19, 14,317	0, 99,320	20, 12,678	0, 6012	62, 8435	0, 185,482	0, 185,482	

MRI: magnetic resonance imaging; CT: computed tomography; SD: standard deviation; CV: coefficient of variation; IQR: interquartile range

**Fig. 1:**
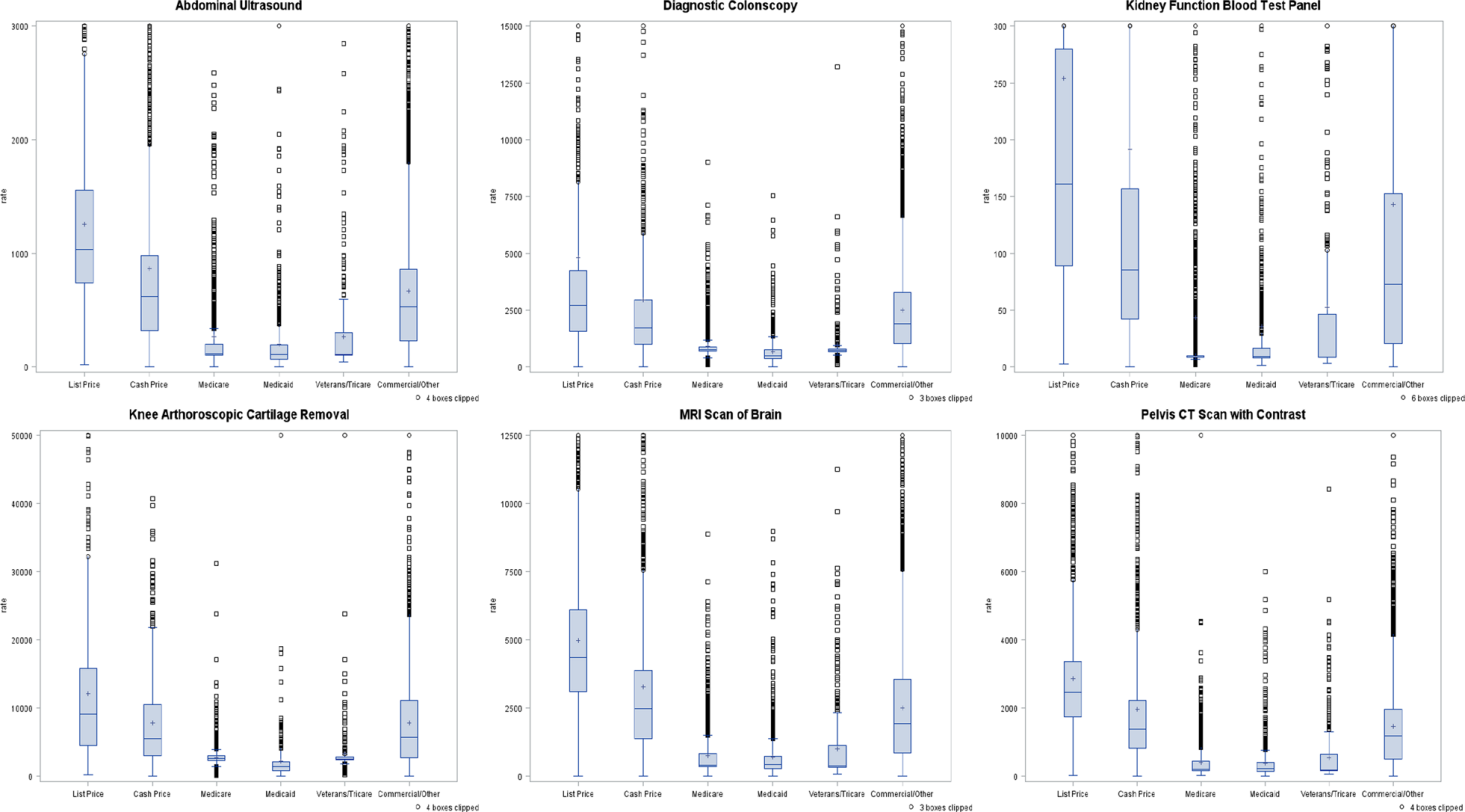
Box plots of prices by type of insurance plan. Extreme values above 10 times the average IQR away from the average 1st quartile across groups were cut off from the plots for readability (i.e. clip factor of 10 was applied)

Table 1 and Fig. 1 demonstrate the marked variability in hospital pricing. The minimum number of prices reported was 18,679 for knee arthroscopic cartilage removal (reported by 908 hospitals with an average of 21 prices/hospital), while the maximum number of prices reported was 44,921 for abdominal ultrasound (reported by 1861 hospitals with an average of 24 prices/hospital).

All six procedures demonstrated statistically-significant variation in pricing by plan type (all *p* < 0.0001); for each procedure, the Medicaid price was the lowest price reported, and the list price was the highest price reported (Table 1). For example, for abdominal ultrasound, reported prices ranged from $0.0 to $42,640.0. The overall mean price was $623.5 (median = $444.0, IQR = $159.9–833.1), and the mean prices by plan type ranged from $198.7 (median = $113, IQR = $70.3–193.4) for Medicaid to $1257.2 (median = $1036, IQR = $738.0–1557.2) for list price, a 6.3-fold difference (*p* < 0.0001).

The CVs for all procedures exceeded 100%, ranging from 106% for knee arthroscopic cartilage removal to 397% for kidney function blood test panel. As illustrated in Fig. 1, the distributions of these prices by plan type demonstrated considerable positive skew. Prices were very heterogeneous with substantial overlap between pricing for all plan types. For example, although Medicaid prices were in general much lower than prices for commercial/other insurances, there were still many records of individual Medicaid prices much higher than the average price for commercial/other insurances for the same procedure.

The procedure with the least variation in pricing by plan type was knee arthroscopic cartilage removal. The overall mean price was $7014.7, and the mean prices by plan type ranged from $2252.1 for Medicaid to $12,104.0 for list price, a 5.4-fold difference (*p* < 0.0001). The procedure with the greatest variation in pricing by plan type was pelvis CT scan with contrast. The overall mean price was $1353.2, and the mean prices by plan type ranged from $382.7 for Medicaid to $2875.7 for list price, a 7.5-fold difference (*p* < 0.0001).

In this analysis of 1982 US hospitals, substantial variability in pricing was observed both overall and by plan type. In general, reported hospital pricing was highest for the list price, followed by cash price and prices negotiated with commercial insurance plans. Government insurance, including Medicare, Medicaid and Veterans/Tricare plans, had much lower prices. However, prices were very heterogeneous with substantial overlap between pricing for all plan types.

The CMS rule requiring that hospitals disclose their pricing was intended to empower patients and drive market competition. However, it is unclear how patients access and use this information in decision making about healthcare. First, it is noteworthy that hospitals reported an average of 23 distinct prices across six plan types for each procedure; this translates into several prices listed per hospital, per plan type, per procedure. It remains to be seen whether patients can identify the pricing relevant to their situation, which will be particularly challenging for patients with low health literacy [6]. Second, considerations beyond price may have large impacts on healthcare decision making. For example, at the time of emergency, patients probably do not pay much attention to cost differences. Patients with lower income might be more sensitive to costs compared to patients with higher income. During the coronavirus pandemic, patients may behave differently than normal times and pay more attention to availability and readiness of the health system. There are also many other factors that can influence the choice, such as transportation, prior experience with the hospital, and reputation of the hospital in the local community, etc.

Similarly, the extent to which the CMS rule increases market competition and ultimately reduces healthcare costs is not yet known as it will take time for the impact to manifest itself fully. There is a growing literature about the impact of price transparency on consumers and providers; however, no definitive conclusion has emerged [7]. A recent scoping review suggests that price transparency may have limited direct impacts on consumer choices but may have a larger impact on provider insurer negotiations [8]. It is likely that the impact is stronger for more homogeneous services such as lab tests and imaging services [9]. The large variations in pricing within each insurance type group and the substantial overlaps in pricing by different insurance plan types observed in this study highlight the potential room for provider-insurer negotiations.

Ultimately, however, it is clear that substantial variation in hospital pricing exists. Recent data indicate that the US spends considerably more of its gross domestic product on healthcare costs, but health outcomes are worse, compared to other developed countries [1]. As a result, many patients avoid or delay accessing healthcare because of concerns about costs [10]. Patients who access healthcare may face financial hardship, financial toxicity, or healthcare-related bankruptcy because of these high costs [11]. Policies, such as the CMS rule mandating hospital disclosure of pricing, may ameliorate these challenges to the US healthcare system.

## Limitations

The study has several limitations. First, not all hospitals provided the pricing of these procedures online in a machine-readable form; therefore, those hospitals were excluded [12]. Second, the prices may be negotiable. As a result, what is disclosed online may not be the actual amount paid (especially for the list or cash price), but could be considered more of an initial charge. However, if patients are supposed to use published online prices to make healthcare decisions, they will only have access to information about these initial charges. Third, a future full study should incorporate additional factors besides insurance plan type that may affect hospital pricing (e.g. geographic location, facility type, area income level, market concentration, and health maintenance organization penetration rate, etc.) into a more comprehensive analysis of hospital pricing variation.

## Supplementary Information

**Additional file 1.** Example Bill.

## Data Availability

The data is publicly available at https://turquoise.health/researchers.

## Abbreviations

CMS: Centers for Medicare and Medicaid Services
CPT: Current Procedural Terminology
CT: Computed tomography
SD: Standard deviation
CV: Coefficient of variation
IQR: Interquartile range
