# Supplementary material for: Large variations in hospital pricing for standard procedures revealed
Source: BMC Res Notes. 2022 Apr 5;15:129. doi: 10.1186/s13104-022-06014-2 (PMC8981177; doi:10.1186/s13104-022-06014-2)
Supplement: Supplementary file 1 — Additional file 1. Example Bill. [file 13104_2022_6014_MOESM1_ESM.docx]

| Hospital Name |  |  |  | Please make checks payable and remit to: | |
| --- | --- | --- | --- | --- | --- |
| Address of the Hospital | |  |  | Hospital Name |  |
| Phone Number of the Hospital | |  |  | Address of the Hospital | |
|  |  |  |  |  |  |
|  |  |  |  |  |  |
| Addressee: |  |  |  |  |  |
| Patient Name |  |  |  |  |  |
| Address of the Patient | |  |  |  |  |
|  |  |  |  |  |  |
|  |  |  |  |  |  |
| Statement Number | Patient Name | Statement Date | Due Date |  |  |
| xxxxxxxx | xxxx xxxx | xx/xx/xxxx | Upon Receipt |  |  |
|  |  |  |  |  |  |
|  |  |  |  |  |  |
| Date of Service | Service Description (CPT) | Charges | Insurance (Medicaid) Adjustment | Insurance (Medicaid) Payment | Patient Balance |
|  |  |  |  |  |  |
|  |  |  |  |  |  |
| xx/xx/xxxx | abdominal ultrasound (76700) | $1,036 | ($923) | ($110) | $3 |
|  |  |  |  |  |  |
|  |  |  |  |  | Please pay this amount: |
|  |  |  |  |  | $3 |
|  |  |  |  |  |  |
| Please contact xxx-xxx-xxxx for billing questions and insurance changes. | | | |  |  |
